# First report of co-infections of Marek's disease virus and chicken infectious anaemia virus in poultry flocks in Nigeria

**DOI:** 10.1016/j.vas.2024.100339

**Published:** 2024-02-13

**Authors:** Adeyinka J. Adedeji, Ismail Shittu, Olatunde B. Akanbi, Olayinka O. Asala, Jolly A. Adole, Philip A. Okewole, Gabriel O. Ijale, Dennis Kabantiyok, Felix Idoko, Johnson J. Shallmizhili, Paul A. Abdu, Shedrach B. Pewan

**Affiliations:** a National Veterinary Research Institute Vom, Nigeria; b Department of Veterinary Pathology, Faculty of Veterinary Medicine, University of Ilorin, Nigeria; c Federal Ministry of Agriculture and Rural Development, Abuja, Nigeria; d Faculty of Veterinary Medicine, Ahmadu Bello University Zaria, Nigeria

**Keywords:** Chicken infectious anaemia virus, Co-infection, Immunosuppression, Marek's disease virus, Nigeria

## Abstract

Marek's disease (MD) and chicken infectious anaemia (CIA) are viral immunosuppressive diseases of poultry caused by the MD virus (MDV) and CIA virus (CIAV) respectively. Despite vaccination against MD, the incidence of the disease in vaccinated poultry flocks in Nigeria persists. However, underlying factors like co-infection with CIAV have not been investigated in the country. This study was designed to investigate possible co-infections of MDV and CIAV in poultry flocks in Nigeria. In 2016, tumorous tissue samples were collected from suspected cases of MD at necropsy in Jos, Plateau State, Nigeria. The samples collected were fixed in formalin for histopathological examination, genomic DNA was extracted from a second part and analysed by polymerase chain reaction (PCR), targeting the *meq* and VP1 genes of the MDV and CIAV, respectively. The histology results revealed that the cutaneous and proventricular lymphomas were characterized by large numbers of mononuclear cellular infiltrates admixed with heterophils. The PCR results revealed that MDV was detected in 66.7% (16/24), CIAV in 45.8% (11/24), and co-infections of MDV and CIAV were detected in 45.8% (11/24) of the samples analysed. In addition, co-infections of MD and CIA were recorded in 100% (6/6) and 27.7% (5/18) of broilers and layer/pullet’ samples respectively. Phylogenetic analysis of the *meq* gene sequences revealed that the Nigerian MDV clusters with very virulent MDV from Egypt and Italy. While, CIAV sequences were genotype II and genotype III and clustered with CIAVs from Cameroon and China. This is the first report of co-infections of MD and CIA in Nigeria.

## Introduction

Due to the ubiquitous nature of Marek's disease virus (MDV) and chicken infectious anaemia virus (CIAV), the viruses have a worldwide spread and are reported as solitary or co-infections along with other poultry pathogens (Gimeno & Schat, 2018). These viruses impact the health of poultry flocks by causing tumours, anaemia, stunted growth, and secondary infections due to immunosuppression (Davidson et al., 2013). Marek's disease (MD), named after the Hungarian pathologist József Marek, is a viral lymphoid neoplastic disease of poultry caused by the MDV (Biggs & Nair, 2012; Brown et al., 2018). MDV is a ubiquitous DNA virus of the genus *Mardivirus* and family *Herpesviridae* (Nair et al., 2020). MD is a serious threat to the global poultry industry as a result of increased morbidity, mortality and condemnation at slaughter, thereby causing reduced productivity and profitability (Morrow & Fehler, 2004). Initially identified as a virus that is capable of efficiently replicating in B cells, MDV has now been isolated in other immune cells such as dendritic cells, natural killers and macrophages (Zhang et al., 2019). Clinical presentations of MD include paralysis of legs and wings, marked reduction in egg production, mortality with neoplastic lesions in multiple organs, and enlarged peripheral nerves which occurs as a result of transformed T lymphocytes in nerves and visceral organs (Gimeno & Pandiri, 2013; Nair et al., 2020). Diagnosis of MD follows a multiple-step process based on clinical signs, gross pathologic lesions, histopathology and molecular assay (Gimeno & Pandiri, 2013) The *meq* gene is the most prominent and type-specific gene of the MDV serotype I associated with viral oncogenicity and virulence (He et al., 2018; Osterrieder et al., 2006). Prevention of MD is by vaccination, MD vaccines have proven to limit the disease progression, but not provide sterile immunity against the virus (Heidari et al., 2022).

Chicken infectious anaemia (CIA) also known as blue wing disease is a viral immunosuppressive and economically important disease of chicks caused by CIAV (Miller & Schat, 2004; Ou et al., 2018). CIAV is an icosahedral, single-stranded circular DNA virus that belongs to the genus Gyrovirus and family Anelloviridae (Rosario et al., 2017). The CIAV has three overlapping open reading frames coding for viral proteins genes namely VP1, VP2 and VP3(Eltahir et al., 2011; Feng et al., 2020). CIAV was first reported in Japan in 1979 as a contaminant in MD vaccines, and it is widespread with reports in most chicken-producing countries of the world (Schat & van Santen, 2020). The specific clinicopathologic signs of CIA are anaemia, pale bone marrow, haemorrhages, atrophy of the thymus, and secondary infections (Orakpoghenor, 2019). In flocks infected by CIAV, growth rates are retarded and mortality is generally between 10−60% (Schat & van Santen, 2020). CIA is transmitted either vertically or horizontally by the oral route (Fatoba & Adeleke, 2019; Miller & Schat, 2004; Sreekala et al., 2020). Diagnosis of CIA is based on clinical signs, histopathology and molecular detection of CIAV (Smyth & Schat, 2013). Although diagnosis of CIA can be complicated by co-infections with other pathogens (Liu et al., 2022).

MDV and CIAV causes severe immunosuppression in poultry resulting in secondary infections and reduced efficacy of vaccinal protection (Hoerr, 2010; Schat & Skinner, 2022; Zhang et al., 2017). CIAV is the most important confounding pathogen in MD outbreaks and the virus has been recovered from MD outbreaks in several countries (Haridy et al., 2009; Liu et al., 2022; Zanella et al., 2001). In Nigeria, poultry farms are reporting increased incidences of MD, despite vaccination against the disease, this needs further investigation (Gimeno, 2004; Owoade et al., 2010). Previously, underlying co-infections with CIAV were thought to be responsible for recurrence and severity of poultry diseases such as infectious bursal disease (IBD) in Nigeria (Adedeji et al., 2016; Owoade et al., 2010).

Several studies have reported detection of CIAV in poultry flocks in different production systems of the country (Adedeji et al., 2016; Oluwayelu et al., 2005; Shettima et al., 2017). However, to date no published reports that investigated MDV and CIAV co-infections in Nigeria. This study was designed to help investigate co-infection of MDV and CIAV in poultry flocks in Nigeria to provide insight on the two pathogens that can help in designing effective control strategies.

## Material and methods

### Study area

The study area was Plateau State, Nigeria, the country shares borders with Benin Republic, Niger Republic, Chad, Cameroon, and the Atlantic Ocean. Plateau State (9.2182° N, 9.5179° E) is located in North Central Nigeria with 17 local government areas (LGA) and population of 3500,000 people. Plateau State is a hub of poultry production due to its clement climate, with temperatures averaging 21 °C-25 °C sometimes dropping as low as 11 °C. The poultry production system in Plateau State consists of backyard and commercial farms with 50–50,000 chickens per farm (Maduka et al. 2015).

### Samples collection

From April to September 2016, tumorous tissue samples were collected from carcasses of chickens (broilers, pullets and layers) suspected of MD which were presented to Veterinary clinics in Jos, Plateau State, Nigeria. The chickens were all from backyard poultry flocks, sample of carcasses from the same flock were pooled together, and samples were collected from 24 poultry flocks. Of the samples collected, one part was placed on ice and the other part was placed in 10% phosphate buffered formalin fixative and stored at the National Veterinary Research Institute (NVRI), Vom and the Department of Veterinary Pathology, University of Ilorin, Nigeria for laboratory investigation respectively. In all, forty-four (44) samples were collected from 24 carcasses consisting of the spleen (*n* = 19), liver (*n* = 16), skin (*n* = 2), Proventriculus (*n* = 1), thymus (*n* = 1), lungs (*n* = 1) and bone marrow (*n* = 4).

### Histopathology

Following postmortem examination of the carcasses, sections of the spleen, liver, skin, proventriculus, and lungs were removed and fixed in 10% buffered formalin. All tissue samples were then embedded in paraffin, sectioned at 5 µm, mounted on charged microscope slides (Menzel, Braunschweig, Germany), dewaxed in xylene and then in graded concentrations of alcohol as previously described (Akanbi et al., 2017). The sections were mounted on clean glass slides, and stained with hematoxylin and eosin (H&E) stains for histopathologic examination using low and high-powered fields of Carl Zeiss camera-mounted binocular microscope.

### Polymerase chain reaction (PCR), PCR product purification and sequencing

DNA was extracted from homogenates of tissue samples using QIAamp® DNA Kits (Qiagen Hilden, Germany) following the manufacturer's instructions. The DNA was initially screened for MDV as previously described by targeting the *meq* oncogene, while detection of CIAV was done by targeting the highly conserved VPI gene (Eltahir et al., 2011: Tian et al., 2011). The gel was then viewed under UV light in a Syngene Bio-imaging system. The PCR products were purified using a QIAquick Purification kit (Qiagen, Germany). The complete *meq* and VP1 genes of MDV and CIAV respectively were sequenced using previously described protocols ((Eltahir et al., 2011; Tian et al., 2011).

### Phylogenetic analysis

Consensus sequences were obtained from both forward and reverse reads using BioEdit. Confirmation of sequence type was carried out using the BLAST tool, (https://blast.ncbi.nlm.nih.gov/Blast.cgi). Phylogenetic trees were constructed using MEGA 11 inferred using the Neighbour Joining method (Tamura et al., 2021). CIAV and MDV sequences were retrieved from the GenBank for the construction of the phylogenetic trees for MDV and CIAV. The sequences were submitted to the gene bank with accession numbers OL804264 -OL804270 for CIAV and OQ129422-OQ129424 for MDV.

## Results

### Necropsy findings and histology results

**Fig 1:**
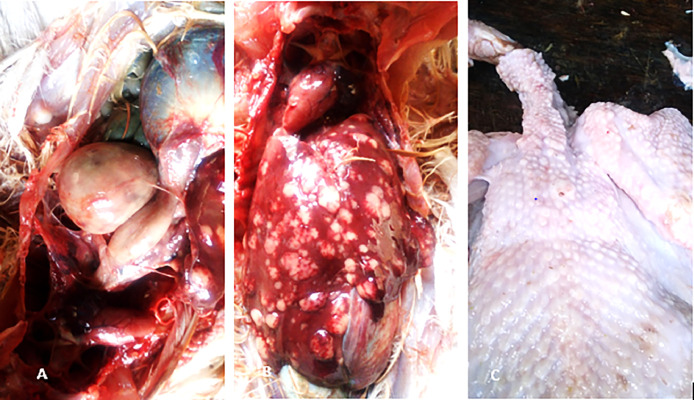
**Gross pathology 1A**: Severe splenomegaly in a 8 weeks old broiler with co-infection of chicken infectious anaemia (CIA) and Marek's disease (MD) confirmed by polymerase chain reaction (PCR). **1B:** Multifocal lymphoma in 13 weeks old pullet with co-infection of CIA and MD confirmed by PCR. **1C**: Cutaneous lymphoma in 9 weeks old broiler with co-infection of CIA and MD confirmed by PCR.

**Fig. 2:**
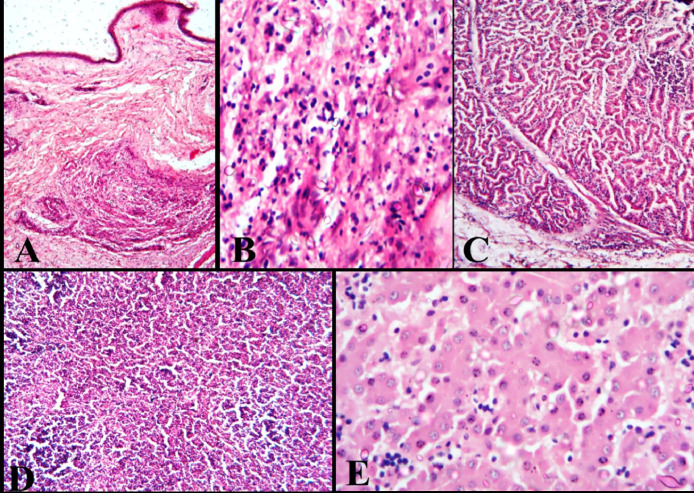
**Histopathology 2A:** Chicken with skin lymphoproliferative nodule, severe focal nodule composed of large mononuclear cellular inflammatory cells within the deep dermis (H&E 100X). **2B**: In the high-powered field of A, the deep dermis nodule is composed mainly of lymphocytes and occasional macrophages and heterophils. There is vasculitis and haemorrhage (H&E 400X). **2C:** Proventricular gland infiltrated moderately by mainly lymphocytes and occasional macrophages and heterophils (H&E 100X). **2D:** spleen densely packed with lymphocytes and occasional macrophages and heterophils with multifocal areas of lymphoid necrosis (H&E 100X). E, liver showing hepatocellular necrosis and intrasinusoidal and interstitial infiltration by lymphocytes with small 2–4 eosinophilic intranuclear inclusions (H&E 400X).

Based on veterinary clinic records, the chickens were from ages 7–52 weeks, consisting of broilers 25% (6/24) and layers 75% (18/24) All the broiler flocks (6) sampled in this study were not vaccinated, while 64.3% (9/14) of the layer flocks were vaccinated against MD with CVI988/Rispens vaccine at day 1 at the hatchery (Table 1). The necropsy examination revealed various pathological manifestations of MD including severely enlarged spleen (11/24) (Fig 1A, Table 1), nodular multifocal lymphoma on the liver (14/24) (Table 1, Fig 1B), cutaneous lymphoma (3/24) (Table 1, Fig. 1C). Other gross pathological lesions observed were prominent proventricular glands, lymphoma on the lungs, stunted growth rate (2/24) and atrophy of the thymus and severe emaciation of some of the carcasses (9/24) (Table 1). The skin of some of the carcasses had lymphoproliferative nodules in severe focal nodules composed of large numbers of mononuclear cellular inflammatory cells within the deep dermis mainly lymphocytes and occasional macrophages and heterophils; there was vasculitis and haemorrhage (Fig. 2A&B). Also, the proventricular glands were interspersed moderately by mainly lymphocytes and occasional macrophages and heterophils (Fig. 2C). The spleen was densely packed with lymphocytes and occasional macrophages and heterophils with multifocal areas of lymphoid necrosis. The liver showed hepatocellular necrosis and intrasinusoidal and interstitial infiltration by lymphocytes.

**Table 1:** History, sample collected, necropsy findings and polymerase chain reaction results of samples collected from poultry in Jos, Plateau State, Nigeria.

Date of collection	Sample Identification number	Specie	Age (Weeks)	Flock history and necropsy lesions	Sample collected	PCR Results	
						CIAV	MDV
30/04/2016	VSD 261	Layer	18	Hepatic lymphoma, Flock vaccinated with CVI988/Rispens vaccine at day 1 at the hatchery.	Liver, spleen	Neg	Pos
12/05/2016	VSD 551	Layer	28	lymphoma on the Liver, tumorous of the visceral organ. MD vaccinated status unknown	Liver, spleen	Neg	Pos
18/05/2016	VSD 563	Layer	52	Lymphoma on the liver and enlarged Spleen. MD vaccinated unknown	Liver, spleen	Neg	Pos
18/05/2016	VSD 565	Pullets	16	Hepatic and splenic lymphomas. MD vaccinated unknown	Liver spleen	Neg	Pos
06/06/2016	VSD 27	Pullets	8	severe emaciation, stunted growth and high mortality. Flock vaccinated with CVI988/Rispens vaccine at day 1 at the hatchery.	bone marrow spleen	Pos	Pos
07/06/2016	VSD 264	Broiler	7	Hepatic lymphoma. Not vaccinated against MD	Liver, spleen	Pos	Pos
10/06/2016	VSD 28	Pullets	12	Lymphoma on the liver and enlarged Spleen. Flock vaccinated with CVI988/Rispens vaccine at day 1 at the hatchery.	Liver, spleen	Pos	Pos
10/06/2016	VSD 29	Broiler	9	Hepatic and splenic lymphomas, not vaccinated against MD	Spleen	Pos	Pos
10/06/2016	VSD 30	Pullets	14	severe emaciation, poor growth rate, high flock mortality. Flock vaccinated with CVI988/Rispens vaccine at day 1 at the hatchery.	Spleen	Pos	Pos
13/06/2016	VSD 33	Layers	23	Hepatomegaly, flock vaccinated with CVI988/Rispens vaccine at day 1 at the hatchery	Liver, spleen	Neg	Neg
14/06/2016	VSD 34	Layers	30	Hepatic and splenic lymphomas	Liver, bone marrow	Pos	Pos
28/06/2016	VSD 37	Pullets	12	severe emaciation, flock vaccinated with CVI988/Rispens vaccine at day 1 at the hatchery	Liver, Spleen	Neg	Neg
28/06/2016	VSD 38	Pullets	20	Hepatomegaly, MD vaccinated status unknown	Liver, spleen	Neg	Neg
30/06/2016	VSD 39	Broiler	10	Splenomegaly, hepatomegaly, emaciation, not vaccinated against MD	Spleen	Pos	Pos
30/06/2016	VSD 41	Pullets	12	Splenomegaly, Hepatomegaly, Emaciation. Flock vaccinated with CVI988/Rispens vaccine at day 1 at the hatchery	Spleen	Pos	Pos
30/06/2016	VSD 43	Pullets	13	Splenomegaly, hepatomegaly, emaciation MD vaccinated unknown	Liver	Pos	Pos
30/06/2016	VSD 46	Pullets	16	Severe emaciation, MD vaccinated unknown	Spleen, liver	Neg	Neg
03/07/2016	VSD 280	Broiler	7	tumour-like cutaneous lesions and hepatic lymphoma, prominent proventricular glands, not vaccinated against MD	Proventriculus, Spleen, Skin, liver	Pos	Pos
03/07/2016	VSD 281	Broiler	8	tumour-like cutaneous lesions and hepatic lymphomas, not vaccinated against MD	Liver, spleen, skin	Pos	Pos
25/07/2016	VSD 47	Pullets	9	Severe emaciation and, Hepatic lymphoma. Flock vaccinated with CVI988/Rispens vaccine at day 1 at the hatchery	Liver, bone marrow	Neg	Neg
04/08/2016	VSD 48	Broiler	9	Hepatic lymphoma, tumour-like cutaneous lesions and splenomegaly, not vaccinated against MD	Liver, skin	Pos	Pos
04/08/2016	VSD 49	Layers	35	Hepatic lymphoma, Flock vaccinated with CVI988/Rispens vaccine at day 1 at the hatchery	Liver, bone marrow	Neg	Neg
14/05/2016	VSD 52	Pullet	14	emaciation and lymphoma on liver, Flock vaccinated with CVI988/Rispens vaccine at day 1 at the hatchery	Spleen	Neg	Pos
28/04/2016	VSD 534	Layer	30	Splenomegaly, Hepatomegaly, flock vaccinated with CVI988/Rispens vaccine at day 1 at the hatchery	Liver, spleen	Neg	Neg

Pos= Positive, Neg=Negative.

### Polymerase chain reaction results

Of the twenty-four samples collected, MDV was detected in 66.7% (16/24), and CIAV was detected in 45.8% (11/24). All CIAV-positive samples in this study were also positive for MDV. While co-infections of MD and CIAV were recorded in 100% (6/6) and 27.7% (5/18) of broilers and layer/pullet samples respectively. Majority of the co-infections were in poultry of ages 7–14 weeks, broilers (7–9 weeks) and pullets (8–14 weeks) (Table 1). Necropsy findings were more severe in poultry with co-infections of MDV and CIAV (Table 1). Necropsy lesions that were common in the co-infection of CIA and MD were severe emaciation (5/11), stunted growth (2/11), hepatomegaly/hepatic lymphoma (8/11), tumour-like cutaneous lesions (3/11) and high flock mortality (2/11) (Table 1).

### Molecular analysis

**Fig. 3:**
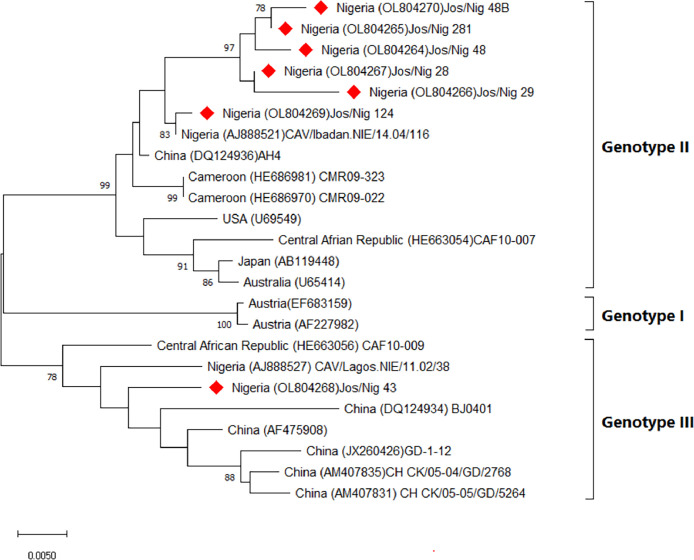
Phylogeny of VPI of Nigeria Chicken infectious anaemia virus. The tree was constructed in MEGA 11 using the neighbour joining at 1000 bootstrap replicates. The evolutionary history was inferred using the neighbour-Joining method. The Nigerian CIAV isolates from this study are highlighted with diamond shape.

**Fig 4:**
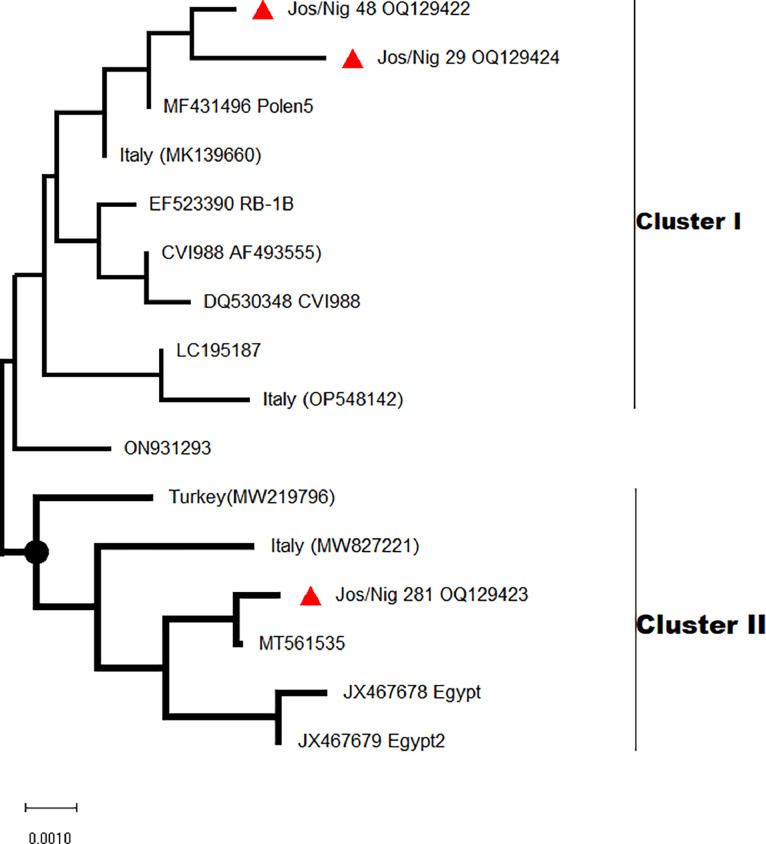
Phylogenetic tree of *meq* oncogene of Marek's disease virus. The tree was constructed in MEGA 11 using the neighbour-joining at 1000 bootstrap replicates. The evolutionary history was inferred using the neighbour-Joining method. The Nigerian MDV isolates from this study are highlighted with triangle shapes.

Seven CIAV sequences were successfully characterized belonging to genotypes II and III. Nigerian CIAVs were similar to viruses from China and Cameroon (Fig 3). Three MDV *meq* gene amplicons were successfully sequenced and phylogeny of *meq* sequences showed they clustered with very virulent MDV and 97–98% similarity with MDV sequences obtained from Turkey and Italy (Fig 4).

## Discussion

Nigeria has the largest population of layer chickens and is the fourth largest producer of broilers in Africa with country's poultry industry being the most commercialized sector of the agricultural industry (Adedeji et al., 2022; Shittu et al., 2019). Due to the importance of poultry production to livelihoods in the country, disease prevention and control is essential. This study confirms for the first time the co-infections of MDV and CIAV in poultry flocks in Nigeria.

Outbreaks of MDV and CIAV have been reported in Nigeria as separate infections in different parts of the country (Oluwayelu et al., 2008; Owoade et al., 2004,; 2008; Okwor & Eze, 2011; Jwander, Abdu, Ibrahim and Nok, 2014; Sani et al., 2017; Shettima et al., 2017) Though, co-infections of CIAV and infectious bursal disease virus had also been reported in the country (Adedeji et al., 2016), that of CIA and MD remained unreported. Co-infection of CIA and MD was confirmed by gross lesions, histopathology, molecular analysis in the current investigations.

The typical gross lesions observed at necropsy in the co-infection of CIA and MD were tumour-like cutaneous lesions, splenomegaly, hepatomegaly, and severe emaciation alongside the history of high flock mortality and stunted growth in some of the poultry flocks (Table 1). Similarly, results from an experimental study reported the severity of gross lesions in visceral organs, tumour-like cutaneous lesions, stunted growth, and high flock mortality in a group of chickens infected with virulent MDV and CIAV compared to groups infected with virulent MDV or CIAV alone (Haridy et al., 2009). Also, other reports support findings from this study that CIAV infection aggravates pathology and severity of MD outcomes (De Boer et al., 1992; Miles et al., 2001; Morrow & Fehler, 2004; Otaaki et al., 1988; Smyth & Schat, 2013).

Several reasons have been expounded for the severity of pathology of MD in co-infections with CIAV. These include lymphoid organ atrophy, suppression of vaccinal response, and MD vaccine failures (Gimeno & Schat, 2018; Haridy et al., 2009; Zhang et al., 2019). Similarly, early mortality syndrome occurs in birds within the first two weeks of age due to dual infection with MDV and CIAV (Haridy et al., 2009). It has also been shown that morbidity and mortality are considerably enhanced in chickens with dual infection of MDV and CIAV, which may be due to virus-induced immunosuppression (Gimeno & Schat 2018; Smyth & Schat, 2013). Usually, when both MDV and CIAV co-infect the same chicken/flock such infections are either evident or concealed affecting the immunological response of affected flocks when vaccinated hsi(Hoerr, 2010). Reports of MD cases in vaccinated and revaccinated flocks may be attributed to co-infection with CIAV (Adedeji et al., 2022). From the histopathology results, the only lesions consistent with CIAV infection was the presence of eosinophilic intranuclear inclusions within hepatocytes of the liver samples which suggests that CIAV was acquired vertically in the younger chickens (Miller & Schat, 2004). While, histologic findings in this study such as skin lymphoproliferative nodules, presence of lymphocytes, macrophages, and heterophils with multifocal areas of lymphoid necrosis in visceral organs with lymphoma is consistent with MD histopathologic lesions (Nair et al., 2020; Smyth & Schat, 2013).

Hatcheries in Nigeria routinely vaccinate layer-type chickens against MD, but not broiler-type chickens (Adedeji et al., 2022). Hence, it is not surprising that the incidence of MD in broilers is on the increase aggravated by the co-infection with CIAV as reported in this study (Adedeji et al., 2022). Moreover, most of the CIA cases were in birds between 7 and 9 weeks suggesting it may be vertically acquired, hence the need to institute measures to screen chicks produced and sold by hatcheries before selling to the public to forestall the continued spread of CIAV. Although, CIA vaccines are commercially available and confer immunity against the disease, however, there is no evidence of their use in Nigeria (Diaz, 2014). Furthermore, global best practices in the poultry industry recommend the vaccination of all commercial poultry including broilers against MD (Diaz, 2014; Schat, 2016). In Nigeria, on the other hand, broilers are not administered MD vaccines, because they are processed earlier or younger, and to reduce production costs. (Adedeji et al., 2022). However, the goal of vaccinating broilers against MD is to prevent transient paralysis syndrome and reduce condemnation at processing associated with skin lesions (Diaz, 2014). CIAV was first reported as a contaminant of MD vaccines and a study in Nigeria also reported Avian leukosis virus J in poultry vaccines (Shittu et al., 2019). With the unregulated importation of poultry vaccines in Nigeria, CIAV as a vaccine contaminant needs to be investigated.

Phylogeny of the sequence data, revealed virulent MDV which clustered around MD viruses from Italy and Egypt belonging to both clusters I and II (Fig 3). A recent study also reported virulent MDV from Nigeria that clusters with MDVs from Egypt and Hungary (Oluwayinka et al., 2023). Likely, these virulent MDVs were introduced into Nigeria poultry flocks via unregulated importation of breeder stock and fertile hatching eggs. In contrast, the CIAVs are diverse belonging to genotypes II and III, similar to CIA viruses from China and Cameroon. Similar genetic studies of CIAV in Nigeria, Cameroon, and the Central African Republic confirmed CIAV genotypes II and III were circulating in poultry in these countries (Ducatez et al., 2006; Snoeck et al., 2012).

## Conclusion

This is the first report of co-infections of MDV and CIAV in Nigeria. Furthermore, the study enhances the understanding of the epidemiology of MD and CIA in Nigeria and answers the question of the increasing severity and prevalence of MD despite vaccination. The co-infection of MDV and CIAV in poultry of different ages raises concerns, particularly because of immunosuppression which may lead to increased susceptibility to secondary infections, loss of condition, reduced productivity, increased mortality, and serious economic losses. Enhanced disease investigation and surveillance coupled with the implementation of biosecurity and vaccination at hatcheries in Nigeria are needed to mitigate the impact of MD and CIA in the country.

## Ethical statement

This study was approved by the National Veterinary Research Institute Animal Ethics Committee Vom, Nigeria (AEC/03/86/20).

## CRediT authorship contribution statement

**Adeyinka J. Adedeji:** Writing – review & editing, Writing – original draft, Methodology, Investigation, Formal analysis, Data curation, Conceptualization. **Ismail Shittu:** Writing – review & editing, Writing – original draft, Methodology, Investigation, Formal analysis, Conceptualization. **Olatunde B. Akanbi:** Writing – review & editing, Writing – original draft, Formal analysis, Data curation, Conceptualization. **Olayinka O. Asala:** Writing – review & editing, Writing – original draft, Formal analysis. **Jolly A. Adole:** Writing – review & editing, Investigation, Formal analysis, Data curation. **Philip A. Okewole:** Writing – review & editing, Conceptualization. **Gabriel O. Ijale:** Writing – review & editing, Investigation, Data curation. **Dennis Kabantiyok:** Writing – review & editing, Writing – original draft, Formal analysis, Data curation. **Felix Idoko:** Writing – review & editing, Writing – original draft, Investigation, Formal analysis. **Johnson J. Shallmizhili:** Writing – review & editing, Investigation, Data curation, Conceptualization. **Paul A. Abdu:** Writing – review & editing, Writing – original draft, Supervision, Conceptualization. **Shedrach B. Pewan:** Writing – review & editing, Writing – original draft, Funding acquisition.

## Declaration of competing interest

The authors declare that they have no known competing financial interests or personal relationships that could have appeared to influence the work reported in this paper.

## References

[bib0001] Adedeji A.J., Sati N.M., Pewan S.B., Ogbu K.I., Adole J.A., Lazarus D.D., Ijiwo S.J., Okpanachi A., Nwagbo I.O., Joannis T.M., Abdu P.A. (2016). Concurrent infections of chicken infectious anemia and infectious bursal disease in 5 weeks old pullets in Jos, Plateau State, Nigeria. Veterinary Sciences: Research and Reviews.

[bib0002] Adedeji A., Abdu P., Akanbi O., Luka P. (2022). Molecular and pathological investigations of Marek's disease outbreaks in vaccinated poultry farms in Plateau State, North Central-Nigeria. Veterinaria Italiana.

[bib0003] Akanbi B.O., Fereidouni S., Taiwo V.O., Starick E., Okewole P.A., Binder A., Heenemann K., Teifke J.P. (2017). Formalin-fixed and paraffin-embedded tissues of chickens are useful for retrospective studies on pathology of H5N1 Highly Pathogenic Avian Influenza Virus (HPAI) outbreaks in Nigeria. Nigerian Veterinary Journal.

[bib0004] Biggs P.M., Nair V. (2012). The long view: 40 years of Marek's disease research and Avian Pathology. Avian Pathology.

[bib0005] Brown A.C., Reddy V.R., Lee J., Nair V. (2018). Marek's disease virus oncoprotein Meq physically interacts with the chicken infectious anemia virus-encoded apoptotic protein apoptin. Oncotarget.

[bib0006] Davidson I., Raibshtein I., Al-Touri A. (2013). Quantitation of Marek's disease and chicken anemia viruses in organs of experimentally infected chickens and commercial chickens by multiplex real-time PCR. Avian Diseases.

[bib0007] De Boer G.F., Jeurissen S.H.M., Noteborn M.H.M., Koch G. (1992). Biological aspects of Marek's disease virus infections as related to dual infections with chicken anaemia virus. 4th International Symposium on Marek's Disease, Amsterdam/Lelystad.

[bib0008] Diaz F.J.T., Diaz FJT, Martinez CG, van d en B erg T., Pena ST, Hauck R. (2014). Vaccination of poultry.

[bib0009] Ducatez M.F., Owoade A.A., Abiola J.O., Muller C.P. (2006). Molecular epidemiology of chicken anemia virus in Nigeria. Archives of Virology.

[bib0010] Eltahir Y.M., Qian K., Jin W., Wang P., Qin A. (2011). Molecular epidemiology of chicken anemia virus in commercial farms in China. Virology Journal.

[bib0011] Fatoba A.J., Adeleke M.A. (2019). Chicken anemia virus: A deadly pathogen of poultry. Acta Virology.

[bib0012] Feng C., Liang Y., Teodoro J.G. (2020). The role of apoptin in chicken anemia virus replication. Pathogens (Basel, Switzerland).

[bib0013] Gimeno I.M. (2004). Marek's disease.

[bib0014] Gimeno I.M., Pandiri A.R., Gimeno IM (2013). Immunosuppresive diseases of poultry.

[bib0015] Gimeno I.M., Schat K.A. (2018). Virus-induced immunosuppression in chickens. Avian Diseases.

[bib0016] Haridy M., Goryo M., Sasaki J., Okada K. (2009). Pathological and immunohistochemical study of chickens with co-infection of Marek's disease virus and chicken anaemia virus. Avian Pathology.

[bib0017] He L., Li J., Zhang Y., Luo J., Cao Y., Xue C. (2018). Phylogenetic and molecular epidemiological studies reveal evidence of recombination among Marek's disease viruses. Virology.

[bib0018] Heidari M., Zhang H., Hearn C., Sunkara L. (2022). B cells do not play a role in vaccine- mediated immunity against Marek's disease. Vaccine: X.

[bib0019] Hoerr F.J. (2010). Clinical aspects of immunosuppression in poultry. Avian Diseases.

[bib0020] Jwander L.D., Abdu P.A., Ibrahim N.D.G., Nok J.A. (2014). Farmers' awareness of marek's disease and biosecurity practices in poultry production in selected states of Nigeria. Nigerian Veterinary Journal.

[bib0021] Liu Y., Lv Q., Li Y., Yu Z., Huang H., Lan T., Zheng M. (2022). Cross-species transmission potential of chicken anemia virus and avian gyrovirus 2. *Infection*. Genetics and Evolution.

[bib0023] Miles A.M., Reddy S.M., Morgan R.W. (2001). Coinfection of specific-pathogen-free chickens with Marek's disease virus (MDV) and chicken infectious anemia virus: Effect of MDV pathotype. Avian Diseases.

[bib0024] Miller M.M., Schat K.A. (2004). Chicken infectious anemia virus: An example of the ultimate host–parasite relationship. Avian Diseases.

[bib0025] Morrow C., Fehler F. (2004). Marek's disease.

[bib0027] Nair V., Gimeno I., Dunn J., Zavala G., Williams S.M., Reece R.L., Hafner S. (2020). Neoplastic diseases. Diseases of Poultry.

[bib0028] Oluwayelu D.O., Todd D., Olaleye O.D. (2008). Sequence and phylogenetic analysis of chicken anaemia virus obtained from backyard and commercial chickens in Nigeria. Onderstepoort Journal of Veterinary Research.

[bib53] Okwor E.C., Eze D.C. (2011). Outbreak and persistence of Marek’s disease in batches of birds reared in a poultry farm located in Nsukka, south east Nigeria. International Journal of Poultry Science.

[bib0029] Oluwayelu D.O., Todd D., Ball N.W., Scott A.N.J., Oladele O.A., Emikpe B.O., Olaleye O.D. (2005). Isolation and preliminary characterization of chicken anemia virus from chickens in Nigeria. Avian Diseases.

[bib0030] Oluwayinka E.B., Otesile E.B., Oni O.O., Ajayi O.L., Dunn J.R. (2023). Molecular characterization and phylogenetic analysis of Marek's disease virus in chickens from Ogun State, Nigeria. Avian Pathology.

[bib0031] Osterrieder N., Kamil J.P., Schumacher D., Tischer B.K., Trapp S. (2006). Marek's disease virus: From miasma to model. Nature Reviews Microbiology.

[bib0032] Orakpoghenor O. (2019). Chicken infectious anemia: Emerging viral disease of poultry—An overview. Comparative Clinical Pathology.

[bib0033] Otaaki Y., Nunoya T., Tajima M., Kato A., Nomura Y. (1988). Depression of vaccinal immunity to Marek's disease by infection with chicken anaemia agent. Avian Pathology.

[bib0034] Ou S.C., Lin H.L., Liu P.C., Huang H.J., Lee M.S., Lien Y.Y., Tsai Y.L. (2018). Epidemiology and molecular characterization of chicken anaemia virus from commercial and native chickens in Taiwan. Transboundary and Emerging Diseases.

[bib0035] Owoade A.A., Iyiola S.H., Oni O.O. (2010). Report of mixed infection of infectious bursal disease and chicken infectious anaemia viruses. Journal of Natural Sciences Engineering and Technology.

[bib0036] Owoade A.A., Oluwayelu D.O., Fagbohun O.A., Ammerlaan W., Mulders M.N., Muller C.P. (2004). Serologic evidence of chicken infectious anemia in commercial chicken flocks in southwest Nigeria. Avian Diseases.

[bib0037] Owoade A.A., Oni O.O. (2008). Molecular detection of Marek's disease virus in some poultry flocks in southwestern Nigeria by polymerase chain reaction. Nigerian Poultry and Science Journal.

[bib0038] Rosario K., Breitbart M., Harrach B., Segalés J., Delwart E., Biagini P., Varsani A. (2017). Revisiting the taxonomy of the family Circoviridae: Establishment of the genus Cyclovirus and removal of the genus Gyrovirus. Archives of Virology.

[bib0039] Sani N.A., Aliyu H.B., Musa I.W., Wakawa A.M., Abalaka S.E., Oladele S.B., Sa'idu L., Abdu P.A. (2017). A nineyear retrospective study of avian neoplastic diseases in Zaria, Kaduna state, Nigeria. S Journal of Veternary Sciences.

[bib0040] Schat K.A. (2016). History of the first-generation Marek's disease vaccines: The science and little-known facts. Avian Diseases.

[bib0041] Schat K.A., Skinner M.A. (2022). Avian immunology.

[bib54] Schat K.A., van Santen V.L. (2020). Chicken infectious anemia and circovirus infections in commercial flocks. Diseases of poultry.

[bib0042] Shettima Y.M., El-Yuguda A.D., Oluwayelu D.O., Abubakar M.B., Hamisu T.M., Zanna M.Y. (2017). Seroprevalence of chicken infectious anemia virus infection among some poultry species in Maiduguri, Nigeria. Journal of Advanced Veterinary and Animal Research.

[bib0043] Shittu I., Adedeji A.J., Luka P.D., Asala O.O., Sati N.M., Nwagbo I.O., Chinyere C.N., Arowolo O.O., Adole J.A., Emennaa P., Abdu P.A. (2019). Avian leukosis virus subgroup–J as a contaminant in live commercially available poultry vaccines distributed in Nigeria. Biologicals : Journal of the International Association of Biological Standardization.

[bib0044] Smyth J.A., Schat K.A., Gimeno IM (2013). Immunosuppressive diseases of poultry.

[bib0045] Snoeck C.J., Komoyo G.F., Mbee B.P., Nakouné E., Le Faou A., Okwen M.P. (2012). Epidemiology of chicken anemia virus in Central African Republic and Cameroon. Virology Journal.

[bib0046] Sreekala S.M., Gurpreet K., Dwivedi P.N. (2020). Detection and molecular characterization of chicken infectious anaemia virus in young chicks in Punjab region of north-western India. Brazilian Journal of Microbiology.

[bib0047] Tamura K., Stecher G., Kumar S. (2021). MEGA11: Molecular evolutionary genetics analysis version 11. Molecular Biology and Evolution.

[bib0048] Tian M., Zhao Y., Lin Y., Zou N., Liu C., Liu P. (2011). Comparative analysis of oncogenic genes revealed unique evolutionary features of field Marek's disease virus prevalent in recent years in China. Virology Journal.

[bib0049] Zanella A., Dall'Ara P., Lavazza A., Marchi R., Morena M.A., Rampin T. (2001). Interaction between Marek's disease virus and chicken infectious anemia virus. Current Progress on Marek's Disease Research.

[bib0050] Zhang Y., Cui N., Han N., Wu J., Cui Z., Su S. (2017). Depression of vaccinal immunity to Marek's disease by infection with chicken infectious anemia virus. Frontiers in Microbiology.

[bib0051] Zhang Y., Tang N., Luo J., Teng M., Moffat K., Shen Z., Yao Y. (2019). Marek's disease virus-encoded MicroRNA 155 ortholog critical for the induction of lymphomas is not essential for the proliferation of transformed cell lines. Journal of Virology.

